# Computer Modeling of Anterior Circulation Stroke: Proof of Concept in Cerebrovascular Occlusion

**DOI:** 10.3389/fneur.2014.00176

**Published:** 2014-09-19

**Authors:** Thanh G. Phan, James Hilton, Richard Beare, Velandai Srikanth, Matthew Sinnott

**Affiliations:** ^1^Stroke Unit, Monash Medical Centre and Stroke and Aging Research Group, Neurosciences Research Unit, Southern Clinical School, Monash University, Melbourne, VIC, Australia; ^2^Mathematics, Informatics and Statistics, CSIRO, Clayton, VIC, Australia; ^3^Developmental Imaging Group, Murdoch Childrens Research Institute, Parkville, VIC, Australia

**Keywords:** leptomeningeal collateral, anatomy, computer modeling, Circle of Willis, stroke

## Abstract

**Background:** Current literature emphasizes the role of the Circle of Willis (CoW) in salvaging ischemic brain tissue but not that of leptomeningeal anastomoses (LA). We developed a computational model of the cerebral circulation to (1) evaluate the roles of the CoW and LA in restoring flow to the superficial compartment of the middle cerebral artery (MCA) territory and (2) estimate the size of the LA required to maintain flow above the critical ischemic threshold (>30% of baseline) under simulated occlusion.

**Methods:** Cerebral vasculature was modeled as a network of junctions connected by cylindrical pipes. The experiments included occlusion of successive distal branches of the intracranial arteries while the diameters of LA were varied.

**Results:** The model showed that the region of reduced flow became progressively smaller as the site of occlusion was moved from the large proximal to the smaller distal arteries. There was no improvement in flow in the MCA territory when the diameters of the inter-territorial LA were varied from 0.0625 to 0.5 mm while keeping the intra-territorial LA constant. By contrast, the diameter of the inter-territorial LA needed to be >1.0 mm in order to provide adequate (>30%) flow to selected arteries in the occluded MCA territory.

**Conclusion:** The CoW and inter-territorial LA together play important supportive roles in intracranial artery occlusion. Computational modeling provides the ability to experimentally investigate the effect of arterial occlusion on CoW and LA function.

## Introduction

Current understanding of the role of the cerebral circulation in stroke is based to a large extent on perfusion studies in cadavers (1, 2) and magnetic resonance (MR) perfusion studies in subjects without cerebrovascular disease (3). However, there are differences between arterial territory maps based on autopsy and MR based perfusion studies versus those based on clinico-radiological correlation studies (4). It has been proposed that these differences may in part be due to the additional protective effect of leptomeningeal anastomoses (LA; the small collateral network, which connects adjacent arteries) (5–8). Despite this, the ability of the LA to function as collateral network is still controversial (9). Understanding the role of the LA is essential in predicting the variation in regional risk of infarction and survival of the ischemic penumbra after arterial occlusion, understanding stroke pathophysiology, and development of therapeutic strategies (10).

### Circle of Willis

Previous research has emphasized the importance of Circle of Willis (CoW) network (primary collateral system) in protecting the brain from the effects of arterial occlusion over the LA network (9, 11, 12). An intact CoW may only re-route flow when a large artery contributing to the CoW (such as the base of the ICA) is occluded. However, the CoW may not offer sufficient protection when the site of occlusion is in the cortical arterial branches distal to the CoW. Further, there are several clinical reports of “sanctuary sites” in the brain, which are relatively protected from the effect of ischemia even in the setting of arterial occlusion (6, 13). These reports suggest that the LA may be of some importance in protecting the brain tissue, arguing for a need to develop better understanding of their role in ischemic stroke.

### Leptomeningeal anastomoses

The *inter-territorial* LA is formed at higher order arterial branches and connects select branches *between* arterial territories middle cerebral artery [MCA to posterior cerebral artery (PCA), MCA to anterior cerebral artery (ACA), ACA to ACA, and ACA to PCA]. Based on cadaver studies, the individual diameters of inter-territorial LA are approximately 0.3 mm (2, 14). By contrast, there is very little description in the literature regarding the functionality of the *intra-territorial LA* connecting the adjacent cortical branches *within* the same arterial territory (within the MCA or ACA or PCA) (2).

Using current imaging methods, we can only infer the existence of LA from the detection of blood flow in that region, but cannot directly visualize the individual anastomosis or estimate LA size and capacity. Computational modeling of the cerebral circulation may provide a more accessible way to study these issues. In this proof of concept study, we describe the application of a three-dimensional (3-D) computational model of the CoW and LA to (1) evaluate the roles of these vessels in restoring flow to the territory of the occluded artery after intracranial artery occlusion and (2) to estimate the diameter of the intra-arterial LA that may be required to preserve cerebral blood flow in the MCA territory.

## Materials and Methods

### Anatomical basis of the computational model

**Figure 1:**
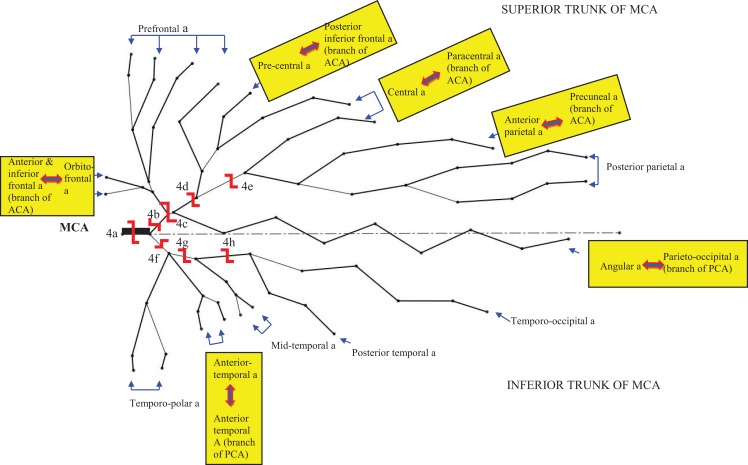
**The branches of the middle cerebral artery (MCA) adapted from Figure 2.19A by Rhoton (15) and Figure 2 by Brozici et al. (9)**. This figure shows an MCA with equal bifurcation into superior and inferior trunks. The anastomoses of the cortical MCA branches are denoted by 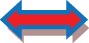. The shape 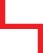 represents the site of simulated occlusion and the capitalized letter corresponds to the description of the site of occlusion in Table 1.

The nomenclature for the branching of the cerebral arteries and diameter and length of the branches in this study were based on authoritative works in the literature (15) (see Supplementary Material). The locations of LA between the branches of the arterial trees were based on the work of van der Eecken (2) and recent reviews (9, 16) (Figure 1).

### Computational modeling

The blood flow within the brain was modeled as a network of junctions or nodes connected by cylindrical pipes (see Supplementary Material). We first created a 3-D model of the major arteries (2, 15) with a computer aided design package (SolidWorks, Solid Works Corp., Concord, MA, USA). The named branches of the MCA, ACA, and PCA were empirically drawn down to the fifth branching order. The LA was represented as connecting pipes between the fifth order branches according to description in the literature. This model was then converted to a list of nodes and pipes, which was transferred into computational software, Matlab, version 5 (The Mathworks Inc., Natick, MA, USA).

We assumed that flow was laminar within the network and formulated a linked set of equations for the system by imposing mass balance and pressure (energy) balance over the network. Inflow and outflow boundary conditions were imposed on the model. We modeled flow in each of the ICA and the basilar artery (BA) (via the vertebral arteries) as coming off the aortic arch and heart (connection below the CoW). Based on previous work (17, 18), the inflow conditions were set to allow 75% of the flow going through the two ICA and 25% through the BA with a pressure condition of 5 kPa was imposed over the outer boundary. The set of equations governing volume flow rate and pressure over the whole network was solved iteratively. The process was repeated for several different experiments in which successive arteries or combinations of arteries were occluded in the anterior circulation. In the model, the outflow is modeled as a consequence of the arterial network branching to smaller and smaller capillaries. We included a drainage component at end points of the arterial branches to accommodate this outflow.

### Experiments

**Figure 2:**
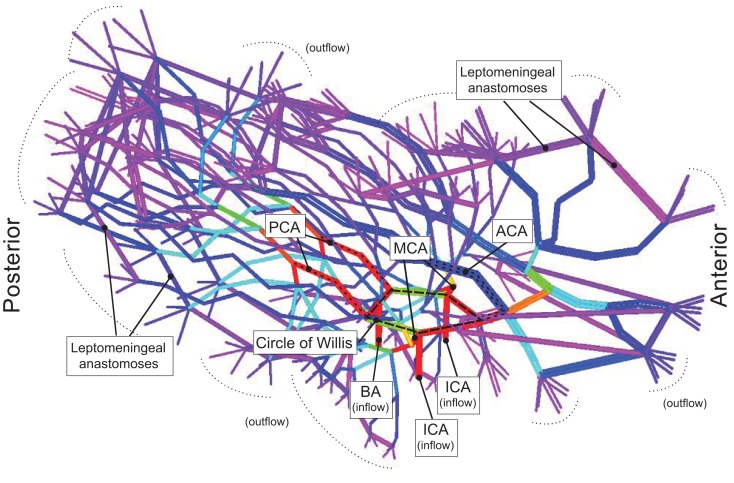
**Model of the Circle of Willis branching into the major arteries and the leptomeningeal arteries is shown**. The color of the branches represents flow rate where red describes high flow rate, and blue describes low flow rate. The Circle of Willis (red) can be seen as the pentagon at the base of the model.

These experiments were based on occlusion patterns encountered in clinical practice as illustrated by examples in Figure 2 (patients with occlusion of the ipsilateral ICA and MCA, the MCA alone, or the cortical branches of the MCA). Figure 1 demonstrates pictorially the occlusive experiments as follows: the first three experiments (1–3) included occlusion of the base of the CoW with and without occlusion of the MCA or the ACA. The remainders involved occlusion of distal branches of the CoW such as the proximal MCA (4a), the superior trunk of the MCA (4b), or its smaller branches (4c–4e), the inferior trunk of the MCA (4f), or its smaller branches (4g–4h). Each experiment was performed under two conditions:

*Condition 1:* Assuming no inter-territorial LA – to evaluate the primary role of the CoW in the setting of intracranial occlusion.
*Condition 2:* To determine the inter-territorial LA diameter required to maintain flow adequate to prevent ischemia (>30% of baseline) (19) in the setting of intracranial occlusion. This process was repeated with the diameters of inter-territorial LA connections varied from 0.25 to 2.0 mm.

## Results

### Condition 1 – setting of no LA

#### Testing the role of CoW in Major Proximal Intracranial Occlusion

In experiment 1 (Table 1), occlusion of the ICA below the formation of the CoW was supplemented by restorative collateral flow from the contralateral ICA and posterior communicating artery (Pcom), and thus flow within the distal branches of the occluded ICA (MCA and ACA) remained normal (100%). From experiment 2 (occlusion of ICA at its base and proximal segment of MCA) to experiment 4a, there was increased flow in the Pcom from 206 to 348.6%, but this did not result in restorative flow into the MCA territory (0%).

**Table 1:** **Effect of experimental arterial occlusion on flow in the right MCA territory (no LA)**.

Site of occlusion			Proximal intracranial occlusion (% flow)				Occlusion of superior trunk of MCA and its branches (% flow)				Occlusion of inferior trunk of MCA and its branches (%)			MCA cortical arteries having more than one stem	MCA cortical arteries with inter-territorial LA

			RICA	ICA + M1	ICA + A1 + M1	M1	Superior trunk	Cortical branch^a^	Cortical branch^b^	Cortical branch^c^	Inferior trunk	Cortical branch^a^	Cortical branch^d^		

Experiment			1	2	3	4a	4b	4c	4d	4e	4f	4g	4h		
Right	P1		100.0	75.1	40.1	75.1	90.0	94.1	95.7	98.2	86.2	93.7	98.1		
	Pcom		100.0	206.0	348.6	206.0	142.6	125.0	118.4	107.8	158.5	126.8	108.0		
	A2		100.0	102.1	92.8	102.1	100.9	100.5	100.4	100.2	101.2	100.5	100.2		
	M1		100.0	0.0	0.0	0.0	59.8	76.4	82.6	92.7	44.8	74.7	92.4		
	Superior trunk of MCA	Orbito-frontal	100.0	0.0	0.0	0.0	0.0	110.2	107.5	103.2	105.4	102.5	100.7	No	Yes
		Prefrontal	100.0	0.0	0.0	0.0	0.0	110.2	107.5	103.2	105.4	102.5	100.7	Yes	No
		Precentral	100.0	0.0	0.0	0.0	0.0	0.0	0.0	115.3	105.4	102.5	100.7	Yes	Yes
		Central	100.0	0.0	0.0	0.0	0.0	0.0	0.0	54.4	105.4	102.5	100.7	Yes	Yes
		Ant. parietal	100.0	0.0	0.0	0.0	0.0	0.0	0.0	0.0	105.4	102.5	100.7	No	Yes
		Post parietal	100.0	0.0	0.0	0.0	0.0	0.0	0.0	0.0	105.4	102.5	100.7	No	No
		Angular	100.0	0.0	0.0	0.0	0.0	0.0	110.3	104.4	105.4	102.5	100.7	No	Yes
	Inferior trunk of MCA	Temp. occipital	100.0	0.0	0.0	0.0	103.9	102.3	101.7	100.7	0.0	0.0	0.0	No	No
		Post temporal	100.0	0.0	0.0	0.0	103.9	102.3	101.7	100.7	0.0	0.0	0.0	No	No
		Mid-temporal	100.0	0.0	0.0	0.0	103.9	102.3	101.7	100.7	0.0	0.0	113.6	No	No
		Ant-temporal	100.0	0.0	0.0	0.0	103.9	102.3	101.7	100.7	0.0	113.1	103.9	No	Yes
		Temp polar	100.0	0.0	0.0	0.0	103.9	102.3	101.7	100.7	0.0	113.1	103.9	No	No
Left		P1	100.0	102.6	134.0	102.6	101.0	100.6	100.4	100.2	101.4	100.7	100.2		
		Pcom	100.0	94.5	−32.1	94.5	97.8	98.7	99.0	99.6	96.9	98.6	99.6		
		A2	100.0	102.0	93.6	102.0	100.8	100.5	100.3	100.1	101.1	100.5	100.1		
		M1	100.0	100.8	98.2	100.8	100.3	100.2	100.1	100.1	100.5	100.2	100.1		

*LA, leptomeningeal anastomoses; ICA, internal carotid artery; M1, proximal MCA; M2, insular part of the MCA; M3, opercular part of the MCA; M4, lateral convexity part of the MCA; M5 distal part of the MCA; A1, proximal ACA; P1, proximal PCA. In experiment 4c, there is occlusion proximal to the second stem of the superior trunk of the MCA^a^. In experiment 4d, there is occlusion of the stem of the superior trunk of the MCA but distal to the origin of the angular artery^b^. In experiment 4e, there is occlusion the superior trunk of the MCA distal to the origin of the precentral artery^c^. In experiment 4f, there is occlusion of the inferior trunk of the MCA. In experiment 4g, there is occlusion of the inferior trunk of the MCA^a^ distal to the origin of the temporo-polar and anterior temporal arteries. In experiment 4h, there is occlusion of the inferior trunk of the MCA^d^ distal to the origin of the mid-temporal artery. The table illustrates the effect of branching pattern of the MCA on flow in simulated occlusion. Note that the prefrontal, precentral and central arteries which have origins from 2 main stems of the superior trunk of the MCA and thus have relatively preserved flow occlusion in experiment 4e. **Flow below 30% are highlighted in red. The cells in blue correspond to the arteries having more than one stem. The cells in gold colour represent those with inter-territorial LA***.

#### Testing the role of CoW in distal intracranial occlusion

In experiment 4b (occlusion of superior trunk of MCA), the compensatory flow from the Pcom was 142.6%, but this was ineffectual in restoring flow to the branches of the superior trunk of the MCA (0%). So, as we moved a site of occlusion more distally in experiment 4c–4g, the region of zero flow became more confined to the territory supplied by the occluded branch (see Table 1).

The results of experiment 4e (occluding the stem for the central, anterior, and posterior parietal arteries) was different from the previous ones. In this case, the flow in the central arteries, which have more than one stem of origin, was 54.4% (reduced only by 45.6%). There was no flow in the anterior and posterior parietal arteries. By contrast, in experiment 4d (occluding the stem for the anterior temporal, precentral, and central arteries and the stem for central, anterior, and posterior parietal arteries), there was no flow in these affected arteries.

#### Condition 2 – testing the role of Intra-territorial LA

With the exception of the findings in the central artery in experiment 4e, data in Table 2 and Figure 4 demonstrate that there was no improvement in flow in the MCA territory even when the diameters of the intra-territorial LA were increased to 2.0 mm while keeping the inter-territorial LA constant (diameter of ≤0.5 mm). The results of these experiments were similar to the setting of no LA.

**Table 2:** **Effect of experimental occlusion. Intra-territorial LA = 2.0 mm, inter-territorial LA = 0.25 mm**.

	Site of occlusion		Proximal intracranial occlusion (% flow)				Occlusion of superior trunk of MCA and its branches (% flow)				Occlusion of inferior trunk of MCA and its branches (%)			MCA cortical arteries having more than one stem	MCA cortical arteries with inter-territorial LA
	
			RICA	ICA + M1	ICA + A1 + M1	M1	Superior trunk	Cortical branch^a^	Cortical branch^b^	Cortical branch^c^	Inferior trunk	Cortical branch^a^	Cortical branch^d^		
	
	Experiment		1	2	3	4a	4b	4c	4d	4e	4f	4g	4h		
Right	P1		211.4	157.9	40.0	75.0	89.9	94.2	95.8	98.4	86.3	93.7	98.1		
	Pcom		−375.3	−147.1	350.0	206.8	143.1	124.6	118.0	107.0	158.8	126.9	108.0		
	A2		90.4	95.0	92.8	102.2	100.9	100.5	100.4	100.1	101.2	100.5	100.2		
	M1		85.4	0.0	0.0	0.0	59.6	77.0	83.1	93.5	45.0	74.8	92.5		
	Superior trunk of MCA	Oribito-frontal	85.4	0.1	0.1	0.1	0.1	108.5	105.9	102.9	105.4	102.5	100.7	No	Yes
		Prefrontal	85.4	0.1	0.1	0.1	0.1	80.2	78.7	105.4	105.4	102.5	100.7	Yes	No
		Precentral	85.4	0.1	0.1	0.1	0.1	10.7	11.9	107.9	105.4	102.5	100.7	Yes	Yes
		Central	85.4	0.1	0.1	0.1	0.1	8.9	10.1	56.2	105.4	102.5	100.7	Yes	Yes
		Ant. Parietal	85.4	0.4	0.4	0.4	0.4	7.6	8.9	13.6	105.4	102.5	100.7	No	Yes
		Post parietal	85.4	0.1	0.1	0.1	0.1	7.3	8.6	13.3	105.4	102.5	100.7	No	No
		Angular	85.4	0.1	0.1	0.1	0.1	5.5	110.3	103.9	105.4	102.5	100.7	No	Yes
	Inferior trunk of MCA	Temp. occipital	85.4	0.0	0.0	0.0	104.0	102.3	101.7	100.6	0.0	0.0	0.0	No	No
		Post temporal	85.4	0.0	0.0	0.0	104.0	102.3	101.7	100.6	0.0	0.0	0.0	No	No
		Mid-temporal	85.4	0.0	0.0	0.0	104.0	102.3	101.7	100.6	0.0	0.0	113.6	No	No
		Ant-temporal	85.4	0.2	0.2	0.2	103.9	102.2	101.6	100.6	0.2	113.1	103.9	No	Yes
		Temp polar	85.4	0.0	0.0	0.0	104.0	102.3	101.7	100.6	0.0	113.1	103.9	No	No
Left		P1	88.5	94.0	134.0	102.6	101.0	100.6	100.4	100.2	101.4	100.7	100.2		
		Pcom	124.9	112.9	−32.7	94.4	97.7	98.7	99.1	99.6	96.9	98.6	99.6		
		A2	91.2	95.5	93.6	102.0	100.8	100.5	100.3	100.1	101.1	100.5	100.1		
		M1	96.3	98.1	98.2	100.8	100.3	100.2	100.1	100.1	100.5	100.2	100.1		

*This table illustrates that increasing the size of the intra-territorial LA makes little difference to flow under intracranial occlusion. **Flow below 30% are highlighted in red. The cells in blue correspond to the arteries having more than one stem. The cells in gold colour represent those with inter-territorial LA***.

#### Condition 2 – testing the role of inter-territorial LA

Data in Table 3 and Figures 4 and 5 showed the diameter of the inter-territorial LA needed to be >1.0 mm in order to provide adequate flow (>30%) to the occluded MCA territory. Under this condition, occlusion of the proximal ICA and MCA (experiment 2), proximal ICA, MCA, and proximal ACA (experiment 3), and MCA occlusion alone (experiment 4a) led to flow below a critical threshold only in selected cortical arteries, which did not possess LA anastomoses (prefrontal, posterior parietal, temporo-occipital, posterior temporal, and middle temporal arteries). In experiments 2–4h, cortical arteries possessing inter-territorial LA had flow >50% whereas those without inter-territorial LA had flow <30%. Importantly, these experiments showed certain arteries, such as the posterior parietal artery, were prone to critically poor flow in the setting of intracranial artery occlusion. Table 3 showed that the diameter of inter-territorial LA is 1.0 mm is sufficient to maintain flow to selected arteries in the territory of the occluded MCA territory.

**Table 3:** **Effect of experimental arterial occlusion. Intra-territorial LA = 0.25 mm, inter-territorial LA = 1.0 mm**.

	Experiment	1	2	3	4a	4b	4c	4d	4e	4f	4g	4h	Arteries having more than one stem	Arteries with inter-territorial LA
	
	Occlusion	RICA	ICA + M1	ICA + A1 + M1	M1	Superior division MCA	branch of superior division MCA^a^	branch of superior division MCA^b^	branch of superior division MCA^c^	Inferior division MCA	branch of inferior division MCA^a^	branch of inferior division MCA^d^		
Right	P1	211.6	160.3	41.8	76.8	90.0	94.3	95.6	98.2	88.0	93.6	98.1		
	Pcom	−385.0	−148.8	362.3	215.6	144.4	125.9	118.7	107.7	165.6	126.0	107.8		
	A2	90.2	96.3	93.9	103.8	102.5	101.0	100.9	100.3	101.1	100.5	100.2		
	M1	85.0	0.0	0.0	0.0	59.3	76.7	82.7	93.0	45.5	75.9	92.8		
	Oribito-frontal	86.5	22.9	22.7	24.5	24.5	108.4	106.2	102.5	104.5	102.0	100.6	No	Yes
	Prefrontal	85.5	7.0	7.1	7.4	7.7	110.1	107.5	103.1	105.3	102.4	100.7	Yes	No
	Precentral	86.6	22.8	22.6	24.3	24.3	23.2	23.2	112.4	104.5	102.0	100.6	Yes	Yes
	Central	86.5	21.5	21.4	22.9	22.9	21.9	21.8	63.8	104.6	102.1	100.6	Yes	Yes
	Ant. parietal	87.7	40.1	39.6	42.8	42.5	41.6	41.6	41.1	103.7	101.7	100.5	No	Yes
	Post parietal	85.8	11.5	11.6	12.3	12.4	11.2	11.1	10.5	105.1	102.3	100.7	No	No
	Angular	86.5	19.9	21.1	21.2	21.4	20.2	109.0	103.6	104.5	102.1	100.6	No	Yes
	Temp. occipital	85.4	5.9	6.2	6.3	104.0	102.3	101.7	100.7	6.0	0.0	0.0	No	No
	Post temporal	85.4	5.9	6.2	6.3	104.0	102.3	101.7	100.7	6.0	0.0	0.0	No	No
	Mid-temporal	85.4	5.9	6.2	6.3	104.0	102.3	101.7	100.7	6.0	0.0	113.5	No	No
	Ant-temporal	87.1	28.4	30.4	30.2	103.1	101.8	101.3	100.6	29.8	109.8	102.9	No	Yes
	Temp polar	85.4	5.9	6.2	6.3	104.0	102.3	101.7	100.7	6.0	112.9	103.9	No	No
Left	P1	88.5	93.7	133.9	102.3	101.3	100.7	100.6	100.2	100.9	100.7	100.2		
	Pcom	125.4	113.9	− 34.8	95.0	96.7	98.4	98.5	99.4	98.6	98.4	99.5		
	A2	91.4	96.5	94.7	103.0	101.9	100.8	100.7	100.3	101.0	100.5	100.1		
	M1	96.4	98.2	98.4	100.9	100.5	100.3	100.2	100.1	100.4	100.2	100.1		

*This table illustrates the importance of inter-territorial LA (at 1.0mm) to flow. Flow below 30% are highlighted in red. **The cells in blue correspond to the arteries having more than one stem. The cells in gold colour represent those with inter-territorial LA***.

#### Illustrated clinical cases

**Figure 3:**
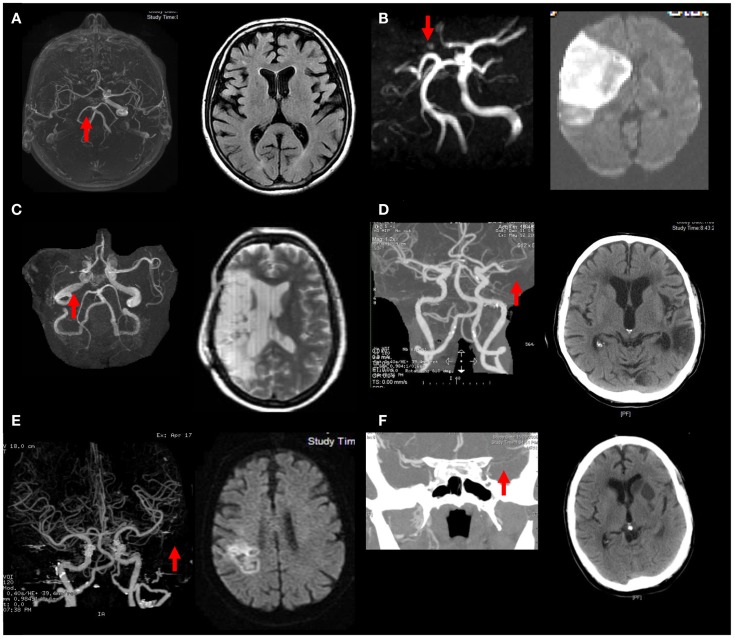
**Illustrations of the relationship between the collateral circulation and infarct size are shown**. **(A)** ICA occlusion: good CoW and LA collateral, no infarct despite; **(B)** ICA and MCA occlusion: poor CoW and LA collateral, large stroke; **(C)** MCA occlusion: poor CoW and LA collateral, large stroke; **(D)** MCA occlusion: good LA collateral, small stroke; **(E)** inferior MCA occlusion: poor LA: moderate sized stroke; **(F)** M4 branch occlusion: good LA collateral, small stroke.

In Figure 3A, where there was internal carotid artery occlusion without associated infarction, it corresponded to the preserved flow findings in experiment 1 (see Table 1). In Figure 3B, where there was internal carotid and middle cerebral arteries occlusion with large areas of infarction, it corresponded to the absence of restored flow findings in experiment 2 (see Table 1). In Figures 3C,D, there was MCA occlusion with two different outcomes: a large infarct (in Figure 3C) and small infarct in the deep compartment (in Figure 3D). The small infarct in case (Figure 3D) would have corresponded to the relatively preserved flow findings in experiment 4a condition 3 (see Table 3). However, this case did not corresponded to the experiment 4a condition 1 where there was no LA collateral. By contrast, the larger infarct in Figure 3C could have occurred because of the absence of restorative flow from the LA collateral, as was the case with experiment 4a condition 1. In Figure 3E, where there was occlusion of the inferior division of the MCA occlusion and consequently a moderate sized infarct in the superficial compartment. It corresponded to the flow findings in experiment 4c condition 1 (see Table 1). In Figure 3F, where there was fourth order branch of the MCA occlusion and small infarct in the superficial compartment, it corresponded to poor flow in a smaller artery territory in experiment 4e (see Table 1).

**Figure 4:**
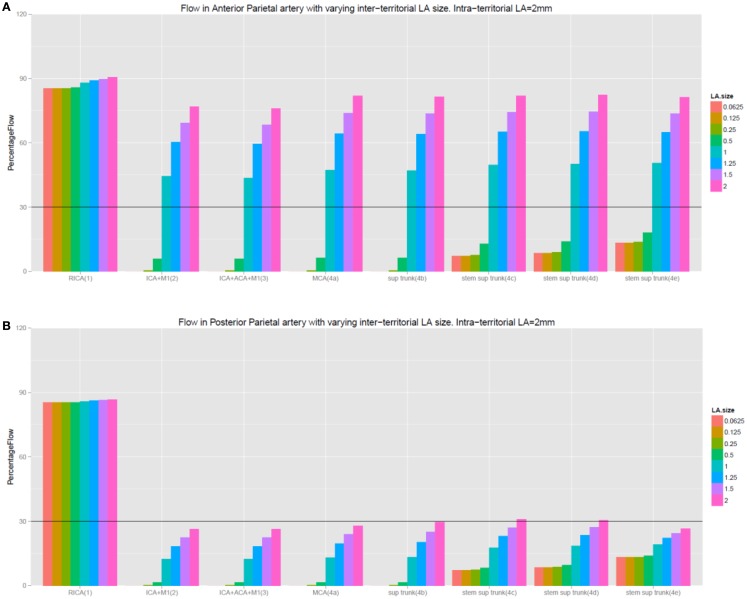
**(A,B)** This figure illustrates that increasing the size of intra-territorial LA does not affect flow under the setting of intracranial occlusion. Inter-LA refers to inter-territorial LA and intra-LA refers to intra-territorial LA. The labeling of the experiments remain the same as in the tables.

**Figure 5:**
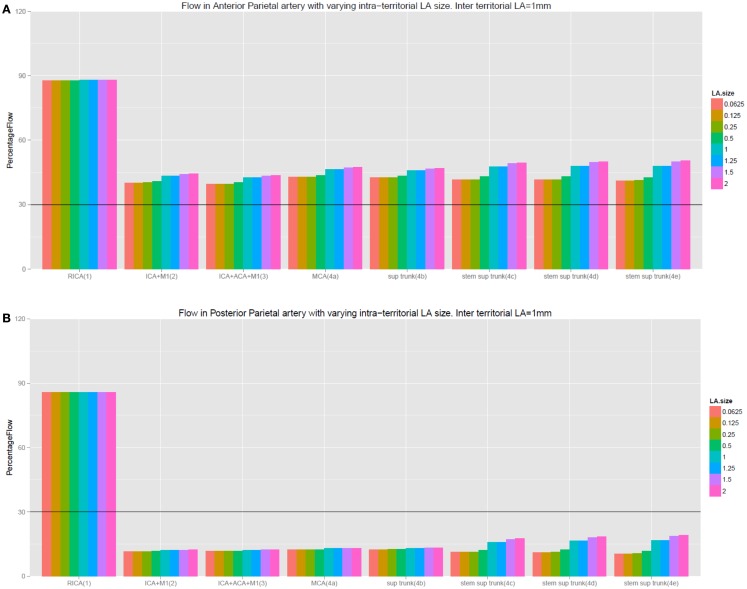
**(A,B)** This figure illustrates the importance of inter-territorial LA to flow under the setting of intracranial occlusion. Observe the difference between Figure 4A (cortical artery with inter-terrorial LA) and **(B)** (cortical artery with no inter-terrorial LA). Although the prefrontal arteries do not possess inter-territorial LA connection, they share the same stem as the precentral artery (which possesses inter-territorial LA). As a result, they benefit from backflow from the precentral artery when the inter-territorial LA is 1.0 in diameter. The labeling of the experiments remain the same as in the table.

## Discussion

We have implemented a computational three-dimensional (3D) model of the cerebral circulation to evaluate the adaptive response of the CoW and LA system in redirecting flow to the territory of the occluded artery. In this proof of concept study, we showed that in presence of experimental intracranial artery occlusion, an intact CoW alone was not sufficient to re-direct flow to the territory of the occluded artery; it required the aid of a functional LA system to restore flow. The intra-territorial LA play a less important role compared to that provided by inter-territorial LA. Importantly, these experiments demonstrated selective vulnerability of arteries which either lack good inter-territorial LA or arise only from a single stem of the MCA. These scenarios mirrored the clinical cases of intracranial occlusion illustrated here as those described in the literature on the beneficial effect of the LA in salvaging the ischemic penumbra (5, 7, 10). Computational modeling provides the ability to experimentally investigate the effect of arterial occlusion on CoW and LA function.

### Adaptive response-variable MCA territory

Based on our experiments, the illustrated cases, and those observations in the literature (4, 20, 21), we proposed that in setting of intracranial occlusion, restoration or cessation flow (Figure 2) to the superficial compartment may occur. We speculate that the former may well suggest a functional LA connecting the ACA and PCA to the cortical branches of the MCA. This may occur as vasodilatory compensatory mechanism. The latter suggest a poorly functioning LA and failure of this compensation. Variability in the LA system may also explain for variability in the arterial territories described by previous investigators (1, 2). These simulation experiments showed that the inter-territorial LA is the key conduit between the arterial territories and that the intra-territorial LA has less important role. The size of the intra-territorial LA was difficult to estimate in these experiments.

Flow in the arteries appeared optimal at inter-territorial LA diameter of 1.0 mm in our simulation experiments. While this finding on inter-territorial LA was larger than those from cadaver studies (2) (0.3 mm), it raises interesting questions on the size of these LA under pathological condition. For example, the LA was shown to increase by as much as 20% in acute stroke (22), 27% after hypoxic preconditioning prior to experimental stroke (23), and even 200% a month after experimental stroke (24). In an autopsy study of patients with Moya Moya (progressive intracranial occlusion and enlargement of LA), the size of the LA can be as large as 1.0 mm (25).

The experiments additionally showed that the complex cortical arterial branching networks could re-route flow to the territory of the occluded artery, particularly the central artery (see Figure 1). This example was demonstrated by the difference in flow in the central and prefrontal arteries as compared with the posterior parietal artery (Tables 1 and 2). In these settings, the preservation of flow was substantial even when LA was lacking. For example, when the fourth stem of the superior trunk was occluded, flow in the central arteries remained at approximately 50% because of additional feed from the third stem of the superior trunk. Because the third stem of the superior trunk gives rise to both the prefrontal and precentral arteries, flow could occur retrogradely through the precentral artery and onto the prefrontal arteries. Thus, regional variations in arterial branching pattern additionally explain some of the differential effect of intracranial arterial occlusion on cortical arterial flow. These scenarios mirror clinical cases presented in Figure 3, the regional variation in infarct risk is published digital probabilistic maps in stroke patients (6) and other clinical case series (4).

Our experiments illustrated the scenarios in which a functional CoW preserved blood flow and support the notion that the anterior communicating artery (Acom) may be crucial in mediating this role of the CoW. This was observed in experiments 1, 2, and 4a, in which the majority of the increased flow was from the contralateral left ICA via the Acom into the distal ACA while bypassing the proximal ACA. In these scenarios, the distal ACA effectively distributed flow to the cortical branches of the ACA and finally onto selected MCA cortical branches via inter-territorial LA. These simulated data are in agreement with the illustrated case in Figure 2. This tandem role of CoW and LA in restoring flow was illustrated in a recent clinical study of ICA and MCA occlusion (26) and are in agreement with those from phase-contrast MR angiographic studies in subjects with ICA occlusion (27) and in modeling experiments on the CoW (11, 28). Our finding and these recent studies de-emphasize the importance of the Pcom in the redistribution of blood flow in the CoW after major proximal anterior intracranial occlusion (12).

A potential limitation of this model was that it was based on a complete CoW and equal division of the MCA into superior and inferior trunks, and there may be other anatomical variants. A complete CoW occurs in 36% and bifurcation of the MCA occurs in 78% of stroke cases (15). It is not possible to display the multiple permutations of the CoW and consequently the corresponding multiple results of occlusions of the intracranial arteries. The purpose here is to provide proof of concept of the adaptive response of the CoW and LA in the setting of intracranial occlusion. Our computational model did not include deep perforating arteries (supplying the deep compartment of the MCA territory) as the intention was to explore the reserve capacity of CoW and LA in restoring flow to the superficial compartment of the brain in the setting of intracranial occlusion. Because the small penetrating arteries such as the lenticulostriate and choroidal arteries do not form anastomosis with each other or the cortical branches of the MCA, then occlusion of the proximal MCA may result in zero flow in these vessels. A further limitation was the reconstruction of the arterial tree without keeping the branching angles at bifurcation points. This oversimplification resulted in some loss of the anatomical geometry of the cerebral vasculature. This issue is less important in the context of our study because we did not intend to study fluid hemodynamics in these experiments, but rather simply to examine the reserve capacity of the CoW and LA. Further, our model makes the assumption that the arteries behave like cylindrical pipes of constant radii. This is known not to be the case in human cerebral circulation, but it is unlikely that a change in the pipe model to one with varying radii will alter the findings of vessels, which would benefit from inter-LA support. A potential weakness of this model is that we have included terms for drainage from the arterial tree but have not directly included named venous structures.

A major strength of our modeling was the creation of a full 3-D model with vascular dimensions derived from authoritative sources (15). To our knowledge, development of a computational model of the entire cerebral circulation has not been attempted previously except in three brain slices (29). A more rigorous method would have been to segment and label different segments of the entire cerebral circulation from whole-brain radiological images, which is an enormous and currently infeasible task. Given the current technology, acquisition of high resolution imaging of the LA and segmentation and labeling of these arteries may be technically extremely demanding. Finally, our experiments do not take into account compensatory blood pressure elevation in acute stroke.

This study showed that intact CoW responds to proximal arterial occlusion to aid flow restoration. When there is interruption of the branches distal to CoW, the inter-territorial LA and arterial tree branching network in selected circumstances can provide significant back up flow in selected arteries under scenarios explored experimentally in this study. This computational model will be further evaluated against patients with documented MCA branch occlusion on CT or MR angiography. In addition, we are looking at the use of computational modeling to explore therapeutic benefits of augmenting the LA and also assist in further studying flow hemodynamics in cerebrovascular occlusion patterns.

## Conflict of Interest Statement

Dr. Thanh G. Phan has received honoraria as speaker for Bayer, Boehringer Ingelheim, Sanofi-Genzyme. He serves on the advisory board for Sanofi received Genzyme for Fabry Disease. The other co-authors declare that the research was conducted in the absence of any commercial or financial relationships that could be construed as a potential conflict of interest.

## Supplementary Material

The Supplementary Material for this article can be found online at http://www.frontiersin.org/Journal/10.3389/fneur.2014.00176/abstract
