# Supplementary material for: Computer Modeling of Anterior Circulation Stroke: Proof of Concept in Cerebrovascular Occlusion
Source: Front Neurol. 2014 Sep 19;5:176. doi: 10.3389/fneur.2014.00176 (PMC4168699; doi:10.3389/fneur.2014.00176)
Supplement: Supplementary file 1 [file Presentation1.PDF]

Supplementary

Circle of Willis

The anterior circulation is supplied by the two internal carotid arteries (ICA) and the posterior circulation is supplied by the basilar artery (BA). The left and right anterior circulations are connected by anterior communicating artery (Acom). The anterior and posterior circulations are connected by the posterior communicating arteries (Pcom). For the purpose of modelling, a complete Circle of Willis was used. A complete CoW has been observed by MR angiography in 36% of normal subjects and 55% of subjects with internal carotid artery (ICA) occlusion [22].

Middle cerebral artery (MCA)

The supraclinoid segment of the ICA gives off the middle cerebral artery (MCA) and the anterior cerebral artery (ACA). The MCA bifurcates into a superior and inferior division. The cortical branches of the MCA supply the superficial compartment of the MCA territory whereas the deep penetrating arteries supply the deep compartment. Unlike the cortical branches, the deep penetrating arteries do not possess anastomoses. In Rhoton’s drawings, some cortical branches such as the prefrontal and central arteries have origins from more than one stem of the MCA [24]. For example (Figure 1), the first major stem from the superior trunk of the MCA gives off the orbito-frontal artery and three branches of the prefrontal arteries. The third stem of the superior trunk gives rise to the fourth branch of the prefrontal arteries and the precentral artery. The prefrontal arteries are therefore privileged in that they share the same stem as the orbito-frontal and the pre-central arteries both of which directly receive inter-territorial LA from the ACA cortical branches. In another example, the two branches of the central arteries have different origins, with one arising from third and the other arising from the fourth major stem of the superior trunk of the MCA. This pattern of branching and

supply from the inter-territorial LA is important when it comes to understanding flow following intracranial arterial occlusion.

Anterior and Posterior cerebral arteries (ACA and PCA)

The A1 segment of the ACA refers to the part proximal to the Acom and the part distal to this is referred as the A2 segment. The ACA gives off the orbitfrontal and frontopolar arteries before giving off two major branches, the callosomarginal and the pericallosal arteries. The first branch of the callosomarginal artery gives off the anterior inferior frontal (AIFA) and middle inferior frontal (MIFA) arteries. The ACA was assumed to have a functional A1 segment and divide into the callosomarginal and pericallosal arteries. The posterior cerebral artery (PCA) was assumed to originate from the BA with functional P1 segment. In this study we used a complete CoW (functional A1 and P1 segments). The diameters and lengths of the branches of the MCA, PCA and ACA were taken from the works of Rhoton and colleagues [24-26, 28]. The P1 segment of the PCA refers to the part proximal to the intersection with the Pcom and the part distal to this is referred as the P2 segment.

The deep compartment (caudate and lentiform nuclei and surrounding white matter tract) is supplied by the lenticulostriate, anterior choroidal, recurrent artery of Huebner and striate branches of the ACA and Acom [29, 30]. These arteries have distinct territories and have very sparse anastomoses. These arteries are not further described in the model.

Leptomeningeal anastomoses of the MCA and ACA

The LA is described between the orbital artery (MCA) and the orbital artery (ACA); orbitofrontal artery (MCA) and the anterior inferior frontal artery (ACA) and frontopolaris artery (ACA); precentral artery (MCA) and the posterior inferior frontal artery (ACA);

central artery (MCA) and paracentral artery (ACA), anterior parietal artery (MCA) and the precuneal artery (ACA); angular artery (MCA) and the parieto-occipital artery (PCA); anterior temporal artery (MCA) and anterior temporal artery (PCA).

Leptomeningeal anastomoses of the ACA and PCA/ACA

The LA is described between the precuneal artery (ACA) and the pericallosal artery (ACA) with the parieto-occipital artery (PCA). The posterior callosomarginal artery (right ACA) with the posterior callosomarginal artery (left ACA); middle portion of the pericallosal artery (right ACA) with the middle portion of the pericallosal artery (left ACA); precuneal branch of the callosomarginal artery (right ACA) with the precuneal branch of the callosomarginal artery (left ACA).

Leptomeningeal anastomoses of the PCA and MCA

The LA is described between the parieto-occipital artery (PCA) and angular artery (MCA) or posterior temporal artery (MCA); anterior temporal artery (PCA) and the anterior temporal artery (MCA).

Computational modelling

The blood flow within the brain was modelled as a network of junctions, or nodes, connected by cylindrical pipes (Figure 2). This model is represented mathematically as a connectivity matrix as described below. We first created a 3-D model of the major arteries using anatomically correct diameter and length measurements [2, 24-26] with a computer aided design package (SolidWorks, Solid Works Corp, Concord, MA, USA). The named branches of the MCA, ACA and PCA were empirically drawn down to the 5th branching order. The LA was represented as connecting pipes between the 5th order branches according to description in the

literature. This model was then converted to a list of nodes and pipes, which was transferred into computational software, Matlab, version 5 (The Mathworks Inc, Natick, MA, USA).

Mass balance was formulated by taking the sum of inflows and outflows to and from a site *i* as the given flow rate at the site *q*:

$$\sum_{\text{inflow}} Q_m - \sum_{\text{outflow}} Q_n = q_i \qquad (1)$$

where $Q_{m,n}$ is the volumetric flow rate over pipes *m* and *n* and q_i is a source or sink at site *i*. Pressure balance is formulated by giving the pressure drop between two sites as the sum of the pressure losses down the pipes connecting the sites:

$$P_i - P_j = \sum_{\text{path}} k_n Q_n \qquad (2)$$

where $P_{i,j}$ is the pressure at sites *i* and *j*. These can be related by a formula for pressure loss as a function of flow rate. We use Poiseuille's law in our model:

$$P_i - P_j = \frac{128\mu}{\pi}\frac{L}{d^4}Q \qquad (3)$$

where μ is the viscosity of blood, taken as 3.5×10^{-3} Pa s, *d* is the diameter of the artery, and *L* is the length of the artery. Arterial diameters in the network which were not known were interpolated from neighbouring pipes.

This system of equations was converted to a matrix and solved using an iterative Newton-Raphson approach. The value at the next iteration, denoted *n+1*, is taken as the value at the current iteration, denoted *n*, plus the incremental change, denoted by Δ:

$$\begin{aligned}\mathbf{Q}^{n+1} &= \mathbf{Q}^{n} + \Delta\mathbf{Q} \\ \mathbf{P}^{n+1} &= \mathbf{P}^{n} + \Delta\mathbf{P}\end{aligned} \qquad (4)$$

where **Q** is a vector with dimension equal to the number of pipes in the system and **P** is a vector with dimension equal to the number of nodes in the system. The iterative increments Δ**P** and Δ**Q** are calculated by solving the matrix:

$$\begin{bmatrix} k_1 & 0 & 0 & \\ 0 & \dots & 0 & \mathbf{A}^T \\ 0 & 0 & k_n & \\ & \mathbf{A} & & 0 \end{bmatrix} \begin{bmatrix} \Delta\mathbf{Q} \\ \Delta\mathbf{P} \end{bmatrix} = -\begin{bmatrix} \mathbf{A}^T\mathbf{P} \\ \mathbf{AQ} \end{bmatrix} - \begin{bmatrix} k_1 Q_1 \\ \cdots \\ k_n Q_n \\ \mathbf{q} \end{bmatrix} \qquad (5)$$

where **A** is the connectivity matrix for the network and **q** is the vector of sources. The connectivity matrix has dimensions of the number of pipes × the number of nodes and contains only zeros, 1 and -1. *k* is a constant given by:

$$k_i = \frac{128\mu}{\pi} \frac{L_i}{d_i^{\,4}} \qquad (6)$$

In practice it was found that this matrix was close to singular and had difficulty converging for small diameter pipes (due to the dependence on d^4). This was resolved by multiplying the entire expression by 1/k, giving:

$$\begin{bmatrix} \begin{matrix} 1 & 0 & 0 \\ 0 & \cdots & 0 \\ 0 & 0 & 1 \end{matrix} & \left\{\begin{matrix} \frac{1}{k_1} & 0 & 0 \\ 0 & \cdots & 0 \\ 0 & 0 & \frac{1}{k_n} \end{matrix}\right\} \mathbf{A}^T \\ \mathbf{A} & 0 \end{bmatrix} \begin{bmatrix} \Delta\mathbf{Q} \\ \Delta\mathbf{P} \end{bmatrix} = -\begin{bmatrix} \frac{1}{k_1} & 0 & 0 & \\ 0 & \cdots & 0 & 0 \\ 0 & 0 & \frac{1}{k_n} & \\ & 0 & & 1 \end{bmatrix} \begin{bmatrix} \mathbf{A}^T\mathbf{P} \\ \mathbf{AQ} \end{bmatrix} - \begin{bmatrix} \mathbf{Q} \\ \frac{1}{k_1} q_1 \\ \cdots \\ \frac{1}{k_n} q_n \end{bmatrix} \qquad (7)$$

The connectivity matrix is formed from a matrix with dimensions of the number of pipes × the number of nodes. The inflow node is marked with a -1 on the matrix and the outflow node is marked as 1, such that there are only two entries per column. Every other location is zero.

$$\begin{matrix} & \textit{pipe} \rightarrow & \\ \mathbf{A} = & \begin{bmatrix} -1 & 0 & -1 & 0 & \cdots \\ 1 & -1 & 0 & -1 & \cdots \\ 0 & 1 & 1 & 1 & \cdots \\ \cdots & \cdots & \cdots & \cdots & \cdots \end{bmatrix} & \begin{matrix} \textit{node} \\ \downarrow \end{matrix} \end{matrix} \qquad (8)$$

The resulting matrix system was solved and iterated using Matlab.

We assumed that flow was laminar within the network, and formulated a linked set of equations for the system by imposing mass balance and pressure (energy) balance over the network. Inflow and outflow boundary conditions were imposed on the model. We modelled flow in each of the internal carotid arteries (ICA) and the basilar artery (BA) [via the vertebral arteries] as coming off the aortic arch and heart (connection below the CoW). Based on previous work [31], [32, 33] the inflow conditions were set to allow 75% of the flow going through the two ICA and 25% through the BA with a pressure condition of 5kPa was imposed over the outer boundary. The set of equations governing volume flow rate and pressure over the whole network was solved iteratively. The process was repeated for several different experiments in which successive arteries or combinations of arteries were occluded in the anterior circulation.

Experiments

The occlusive experiments are described only for right sided intracranial arteries of the anterior circulation. These experiments were based on occlusion syndromes encountered in clinical practice as illustrated by examples in Figure 3 (patients with occlusion of the ipsilateral ICA and MCA, the MCA alone, or the anterior branches of the MCA). Figure 1 demonstrates pictorially the occlusive experiments as follows: The first three experiments (1-3) included occlusion of the base of the CoW with and without occlusion of the MCA or the ACA. The rest involved occlusion of distal branches of the CoW such as the proximal MCA (4a), the superior trunk of the MCA (4b) or its smaller branches (4c-4e), the inferior trunk of the MCA (4f) or its smaller branches (4g-4h). Each experiment was performed under two conditions:

Condition 1: Assuming no intra-territorial or inter-territorial LA - to evaluate the primary role of the Circle of Willis in the setting of intracranial occlusion.

Condition 2: To determine the LA diameter required to maintain flow adequate to prevent ischemia (>30% of baseline) [34] in the setting of intracranial occlusion, the diameters of intra-territorial LA connections were varied from 0.0625 to 2.0mm while keeping the diameter of the inter-territorial LA constant. This process was repeated with the diameters of inter-territorial LA connections varied from 0.0625 to 2.0mm while keeping the diameter of the intra-territorial LA constant.
